# Student experiences of simulation-based learning and its impact on their performance in Objective Structured Clinical Examination in Pediatrics - A mixed method study

**DOI:** 10.12669/pjms.39.4.7287

**Published:** 2023

**Authors:** Sana Saeed, Azam Afzal, Farah Khalid, Fyezah Jehan

**Affiliations:** 1 Sana Saeed, FCPS Assistant Professor, Department of Pediatrics & Department for Educational Development. The Aga Khan University Karachi, Karachi, Pakistan; 2 Azam Afzal, MHPE Senior Manager, Department of Community Medicine & Department for Educational Development. The Aga Khan University Karachi, Karachi, Pakistan; 3 Farah Khalid, MSc Instructor, Department of Pediatrics, The Aga Khan University Karachi, Karachi, Pakistan; 4 Fyezah Jehan, FCPS Associate Professor, Chair, Department of Pediatrics. The Aga Khan University Karachi, Karachi, Pakistan

**Keywords:** Education, learning, simulation training, medical students, clinical skills

## Abstract

**Objective:**

This study aims to assess the effectiveness of integrating simulation for teaching pediatric clinical examination skills to undergraduate, MBBS Year-IV medical students at The Aga Khan University, Karachi, Pakistan.

**Methods:**

In this mixed method study, the Objective Structured Clinical Examination (OSCE) scores of the students who were taught using simulation (batch 2017-2018) were compared with the batch of the academic year 2016 -2017 (taught via traditional methods). In order to explore the experiences of the intervention group, a questionnaire with four open-ended questions was administered at the end of the clerkship.

**Results:**

Students who were taught by simulation, scored significantly higher on the clinical skills stations in Objective Structured Clinical Examination, than in the pre-intervention group (p-value <0.01). The students quoted safety of the learning environment, opportunities for deliberate practice, debriefing and facilitation skills maximized quoted that their experience of learning through simulation, however, some challenges were identified for future improvement.

**Conclusion:**

The result of the study suggested simulation as a useful instructional strategy for teaching examination skills to students in their early years. The student gained confidence through deliberate practice and feedback without compromising patient safety, which translated into improved performance in the high-stakes OSCE examination at the end of the clerkship.

## INTRODUCTION

In conventional medical teaching, patients are an educational resource and have served well to improve the teaching of clinical skills to medical students and trainees such as history taking, communication, examination, and procedural skills.^1^ This skill-based learning is considered problematic due to concerns related to patient safety and wellbeing, accessibility to appropriate patients, and inconsistency among individual clinical teachers.^2^ Patients with clinical findings may be too ill to contribute to bedside teaching.

Other reasons like a shorter hospital stay, a decrease in the number of faculty teachers, increase in the number of students all demand that other media of teaching, such as simulation be integrated into the curriculum.^3^ Simulation-based medical education (SBME) has the advantage of decreasing bedside time, found in one study to be as low as 2.5 minutes compared to the time spent in the classroom (69 minutes).^4^ It also provides an opportunity to do direct observation and give timely feedback to the learners, which is a prerequisite for effective learning of clinical skills. SBME provides a safe environment that facilitates students to practice clinical skills (examination skills, procedures, and counseling) in a simulated setting prior to performing these skills on real patients.^5^

SBME has been shown to improve clinical skills both in novices and experts.^6^ Students also feel more comfortable and encouraged in learning through their mistakes in a simulated situation. Numerous boot camps in various academic programs using low to high-fidelity simulation have equipped novice learners with basic skills. These camps have demonstrated instantaneous increases in self-reported comfort and confidence of interns and new residents in performing technical skills.^7^ The Pediatric specialty is unique in many ways from other fields of medicine, including the varying spectrum of illnesses and their presentation across ages, different needs than adult, distinctive diagnoses, and the need for building connection with children.

It is vital for medical students to learn not only examination skills but also to develop rapport with pediatric patients. There is a paucity of literature focusing on the role of simulation in pediatrics for learning of these skills. The Pediatric clerkship at Aga Khan University (AKU) is an eight week rotation in which Year-IV medical students rotate in five groups (of 20 students each) every academic year. Strategies such as bedside teaching during ward rounds, clinics, faculty lectures and small group learning (tutorials) are employed. During ward rounds, a student may prepare and present one or more patients for their history, verbalize clinical examination findings and inform investigations and management plan to attending faculty.

The supervising faculty does not directly observe the interaction with the patient. After the student presentation, the attending occasionally gets time to demonstrate a relevant clinical skill on real patients, but students don’t really get a chance for demonstration, hampering learning, practice on the same patient and obtaining feedback for improvement. There are faculty-based factors which impact student learning including faculty engagement for teaching and time available for providing feedback, all critical to optimize learning. Concomitant patient care responsibility, family counseling and clinics all create a challenge for the teaching faculty to focus on individualized learning of all students that is of immense importance at this stage.

Simulation based teaching has been integrated in the undergraduate pediatric clerkship in AKU from 2016-2017 with the aim of providing a safe learning environment where medical students observed teaching faculty demonstrating clinical skills, practice on manikins, perform the relevant skills and received feedback. The purpose of this study was to see the effectiveness of simulation-based integration for teaching of clinical skills on student learning, their performance in their Objective Structured Clinical Exams (OCSE) exams and their experiences of learning in that environment as learners.

## METHODS

The study was conducted from November 2017 to December 2019 at the Department of Pediatrics and Child Health, The Aga Khan University (AKU). It was a mixed methodology study where concurrent triangulation was done and the quantitative arm was based on quasi experimental approach.

### Ethical Approval:

It was obtained from the Ethical Review Committee of AKU (5136-DED-ERC17).

Pediatric clerkship rotation has traditionally been using bedside teaching for clinical skills. The pre-intervention group rotating between 2016- 2017(n=103) before the implementation of simulation was considered as having received Bedside method (BM) while the group rotating between 2017-2018 (n=100) received SBM, was taken as post-intervention (SBM) group.

### Simulation-based Intervention:

Six clinical examination skills were selected for teaching through simulation, based on their importance in pediatrics. These included child anthropometry, pediatric cardiovascular, abdominal, respiratory, general physical and lower motor examination. Each of the sessions was of two hours duration. Each group of twenty students was subdivided in two small groups of about 10 students each. The session planner was shared to the students via Moodle by the program administrator.

Two trained facilitators were dedicated to take each session throughout the year, to ensure standardization between two groups. The manikins which were utilized were Mega Kid code by Laerdel and Harvey. After pre-briefing, the students were given a unique theme in each session e.g., my child is not getting tall- Anthropometric examination. The relevant systemic examination theme was first explained and demonstrated by the facilitator and then the students were given the opportunity to have deliberate practice which was followed by re-demonstration and debriefing.

### Data Collection and Analysis:

The scores of the pre-intervention (BM) and SBM groups were extracted anonymously for all end of clerkship rotations (occurring every two months), for a total of five OSCE exams in each of the pre-intervention BM and the post-intervention SBM groups. An open-ended questionnaire was developed comprising of four questions to collect the experiences of medical students about simulation-based teaching of clinical skills in terms of the strengths and areas which needed improvement. The anonymous questionnaire was administered immediately after the OSCE exam. Quantitative data was analyzed using STATA 15.0. The responses of individual questions were transferred on a separate document for analysis. Coding was done independently which contributed to the trustworthiness of data.

## RESULTS

### Quantitative Result:

Total number of students was 203 with male predominance (n=119 (58%)). In the pre-intervention group, the highest average score was reported for abdominal examination skills (82.6 ± 10.8) while the lowest average score was documented for cardiovascular examination. Highest and lowest scores for the post intervention group were found to be at respiratory examination (84.7 ± 10.6) and abdominal examination (70.3 ± 1.7) respectively. The post intervention (SBM) group scored significantly higher as compared to the pre-intervention intervention (BM) group on all the clinical examination skills OSCE stations (p value <0.005) except anthropometric examination (Table-I).

**Table-I:** Pre and Post Innervation Examination Results Comparison.

#	Skills Theme	Pre-intervention Group (BM)	Post-intervention Group (SBM)	Difference in Means (Mean ± SE)	P-value

		Mean ± SD	Mean ± SD	Mean ± SD	
1	Abdominal Examination “ A child with Jaundice”	82.6 ±10.8 n = 103	70.3 ±14.2 n = 100	-12.3 ±1.7	<0.001
2	Cardiovascular examination “ A cyanotic child”	58.0 ±10.9 n = 59	72.7 ±15.2 n = 100	14.70 ±2.0	<0.001
3	Neurological examination “ A child with one sided body weakness”	70.0 ±11.7 n = 103	75.2 ±10.4 n = 81	5.20 ±1.6	0.002
4	Respiratory examination “ A child with breathlessness”	67.8 ±9.1 n = 42	84.7 ± 10.6 n = 100	16.93 ±1.7	<0.001
5	Anthropometric examination “ A child with short stature”	78.5 ±11.4 n = 43	77.6 ±11.1 n = 59	0.93 ±2.2	0.68

In stratified scores comparison by gender (Table-II), both male and female scored higher in intervention group for cardiovascular and respiratory examination while for abdominal examination, the average score noted to be high in pre intervention group, however no significance difference was noted in anthropometric examinations.

**Table-II:** Stratified by gender: Pre and Post Innervation Examination Results Comparison.

Gender	Pre-intervention Group (BM) (Mean ± SE)	Post-intervention Group (SBM) (Mean ± SE)	Difference in Means (Mean ± SE)	P-value
	*Abdominal Examination*			
Male	81.1 ±1.5	70.1±2.2	-10.9±2.6	0.000
Female	84.0±1.4	70.3± 1.8	-13.7±2.3	0.000
	*Cardiovascular Examination*			
Male	57.2±1.8	69.8±2.0	12.5±2.9	0.000
Female	58.8± 2.1	74.8±2.1	16.0±3.3	0.000
	*Neurological examination*			
Male	70.4±1.6	73.8±2.0	3.3±2.6	0.202
Female	69.5 ±1.6	76.1±1.3	6.6±2.1	0.002
	*Respiratory examination*			
Male	69.8±1.6	83.0±1.8	13.2±2.8	0.000
Female	65.5±2.2	85.9 ± 1.2	20.4±2.4	0.000
	*Anthropometric examination*			
Male	76.4±2.5	77.4±2.3	0.98±3.4	0.776
Female	80.7±2.3	77.7± 1.8	-2.9±2.9	0.317

In stratified comparison by Continuous assessment, students with CA score ≥ 50 scored significantly higher in cardiovascular and respiratory examination while students with CA score < 50 scored higher only in respiratory examination. (Table-III)

**Table-III:** Stratified by Continuous assessment score: Pre and Post Innervation examination Results Comparison:

Continuous assessment score	Pre-intervention Group (BM) (Mean ± SE)	Post-intervention Group (SBM) (Mean ± SE)	Difference in Means (Mean ± SE)	P-value
	*Abdominal Examination*			
CA < 50	82.0 ± 1.37	91.0±3.4	8.98 ± 5.8	0.127
CA ≥ 50	83.6 ± 1.6	69.3± 1.4	-14.2 ± 2.5	0.000
	*Cardiovascular Examination*			
CA < 50	59.5±1.8	60.7±3.9	1.1 ± 5.7	0.846
CA ≥ 50	55.5± 2.1	73.2± 1.5	17.6 ± 3.3	0.000
	*Neurological examination*			
CA < 50	68.1± 1.3	78.5±5.5	10.3 ± 5.8	0.082
CA ≥ 50	73.8±2.0	75.0 ± 1.1	1.2± 2.2	0.593
	*Respiratory examination*			
CA < 50	66.5± 1.7	90.7± 3.3	24.1± 4.7	0.000
CA ≥ 50	70.6 ± 2.3	84.4±1.0	13.8± 3.0	0.000
	*Anthropometric examination*			
CA < 50	---	---	---	---
CA ≥ 50	76.5±2.1	77.7±1.45	1.1± 2.7	0.663

### Qualitative Results:

The open-ended questionnaire was responded by all students (n=100) in the post intervention group. Content analysis of the responses yields three key themes, learning through simulation, role of facilitation, way forward for individualized learning. Students considered hands on experience during simulation-based learning of clinical skills a vital contributing factor to their learning. They also appreciated the impact of feedback and safety of learning environment in improving their clinical skills. Below are the few extracts from student responses. “I believe that the hands-on experience helped me a lot during my OSCE exams. I felt that I had the examination scheme prepared in my mind due to repeated exposure and feedback to the same situation”.

Almost all the students emphasized on the importance of having a motivated and engaged facilitator conducting these sessions. They felt faculty involvement is vital to maximize their learning. Students threw some light on highlighting the important role of facilitator in establishing the fiction contract. They reported that they were able to see the manikin differently. Student C: “The way the facilitator demonstrated rapport building, it felt as if she is talking to the real patient.”

The students gave certain suggestions to improve these sessions in term of structure and planning. They felt there should be fewer participants in each group so that each student would have appropriate exposure with the simulator and faculty. They also felt that each session should be focused on one examination skills only so that there is less information to handle. Student V: “Sometimes I felt there is too much to understand and do in one session. It would be great if the sessions are split”

## DISCUSSION

In Pakistan, training of undergraduate medical students follow the conventional system, where the students generally start learning clinical skills in third year, focusing mostly on opportunity-based patient encounters without having any simulation-based training.^8^,^9^ In a study conducted by Sarwar et al on perceptions of medical students regarding bedside learning, multiple aspects were found to be in need of improvement.^10^ These included few opportunities to practice clinical skills, inadequate space for observation, and absence of feedback.

This correlates with the deficits in clinical skills commonly seen in medical and postgraduate students nowadays.^8^ Lack of supervision is also a significant factor; students often interact with patients independently and are not observed by faculty. Thorough faculty supervision is key to almost every aspect of medical education, including competence, communication, and observation of ethics.^11^ Our study reported significant differences in the performance of medical students having exposed to simulation pedagogy. This is in agreement with Shah et al,^12^ where simulation-based learning was introduced for teaching normal vaginal delivery to 3^rd^ year medical students.

The results revealed effect of simulation on learning of skills of medical students, mean score of 8.91±3.20 compared mean of 5.67±1.84 with p<0.01. Mirza et all also the benefits of utilizing integrated clinical skills sessions that the enhanced confidence, motivation, and be effective in achieving learning.^13^ Furthermore, deliberate and repetitive practice allowed the students to rectify errors and improve performance, which facilitates the progression from novice to expert and ultimately reduces the chances of skills decay, as noted by Agha et al.^14^

Considering the under-5 mortality rate in Pakistan (65 deaths per 1000 live births),^15^ the Paediatrics clerkship is a vital component of the MBBS program. It is our key responsibility to maximize the learning experience and motivate students to pursue this specialty in the future.

Supervision, opportunity to practice, and feedback were maximized to enhance their motivation, which is reflected by their full participation in each session during the rotation as well as their end-of-clerkship OSCE. Students appreciated the simulation pedagogy for learning of examination skills.

They verbalized the feedback helped them to improve upon their skills which ultimately boosted their confidence when they performed those skills on real patients and/or in their OSCE. Students termed the simulation-based learning environment as ‘relaxing’, ‘exciting’, and ‘fun’. Similar findings were reported by Au et al, who stressed upon establishing a safe learning environment during simulation-based learning as anxiety could inhibit both performance and perception of learning.^16^

However, this study also found that contact opportunity with the simulator for hands-on practice was occasionally hampered due to the large number of students in each learning group. One solution is increasing the number of facilitators, but holistic faculty development is required to maintain the quality and standard delivery of content and coaching across all groups. To the best of our knowledge this is by far the first study that capture the effect of simulation-based teaching on pediatric examination skills in our local context.

### Limitations:

The data was collected from a single center and may not represent findings in other institutions. Secondly, it utilized a quasi-experimental design due to the structure of Paediatrics clerkship. At the same time, our study found multiple factors that further enhanced the simulation-based learning experience from the perspective of the students.

## CONCLUSION

Using simulation for teaching pediatric clinical examination skills contributed to student’s satisfaction and improvement in student’s performance in high stakes exams. Though the results of this study are significantly encouraging but warrant the need of follow-up study to explore the ultimate impact of intervention on bedside performance of our medical students.

### Authors’ contribution:

**Sana Saeed (SS):** contributed to the conception, design, acquisition of data, interpretation of data, and drafting of the content. She is also responsible for the integrity and accuracy of the study.

**Azam Afzal (AA):** contributed to the design, interpretation, and final approval of the content.

**Farah Khalid (FK):** contributed to data acquisition and interpretation.

**Fyezah Jehan (FJ):** contributed to data interpretation and final approval.

## References

[1] Janicik RW, Fletcher KE (2003). Teaching at the bedside:a new model. Med Teach.

[2] McGraw RC, O'Connor HM (1999). Standardized patients in the early acquisition of clinical skills. Med Educ.

[3] Nair BR, Coughlan JL, Hensley MJ (1997). Student and patient perspectives on bedside teaching. Med Educ.

[4] Tremonti LP, Biddle WB (1982). Teaching behaviors of residents and faculty members. J Med Educ.

[5] Nestel D, Roche J, Battista A (2017). Creating a quality improvement culture in standardized/simulated patient methodology:The role of professional societies. Adv Simul (Lond).

[6] Khan K, Pattison T, Sherwood M (2011). Simulation in medical education. Med Teach.

[7] Esterl RM, Henzi DL, Cohn SM (2006). Senior medical student “Boot Camp”:can result in increased self-confidence before starting surgery internships. Curr Surg.

[8] Martins RS, Sabzwari S, Iqbal M (2021). Effectiveness of simulation-based clinical skills training for medical students in respiratory medicine:A pilot study. J Coll Physicians Surg Pak.

[9] Farooka MW (2018). Demotivating Factors among Third Year Medical Students in a Public Medical College in Pakistan. J Coll Physicians Surg Pak.

[10] Sarwar S, Aleem A, Nadeem MA (2020). Bed side teaching:Student's perception and its correlation with academic performance. Pak J Med Sci.

[11] Keshavarzi M H, Azandehi S.k, Koohestani H (2022). Exploration the role of a clinical supervisor to improve the professional skills of medical students:a content analysis study. BMC Med Educ.

[12] Shah N, Baig L, Shah N, Hussain R, Aly SM (2017). Simulation based medical education;teaching normal delivery on intermediate fidelity simulator to medical students. J Pak Med Assoc.

[13] Mirza MB, Sulaiman A, Hashmi S, Zaki S, Rehman R, Akbar R (2021). Use of simulation based technology in pre-clinical years improves confidence and satisfaction among medical students. J Pak Med Assoc.

[14] Agha S (2019). Effect of simulation based education for learning in Medical Students:A mixed study method. J Pak Med Assoc.

[15] “Mortality rate, under-5 (per 1,000 live births) - Pakistan” World Development Indicators.

[16] Au ML, Lo MS, Cheong W, Wang SC, Van IK (2016). Nursing students'perception of high-fidelity simulation activity instead of clinical placement:A qualitative study. Nurse Educ Today.

